# Profiling of RNA ribose methylation in *Arabidopsis thaliana*

**DOI:** 10.1093/nar/gkab196

**Published:** 2021-03-30

**Authors:** Songlin Wu, Yuqiu Wang, Jiayin Wang, Xilong Li, Jiayang Li, Keqiong Ye

**Affiliations:** Key Laboratory of RNA Biology, CAS Center for Excellence in Biomacromolecules, Institute of Biophysics, Chinese Academy of Sciences, Beijing 100101, China; University of Chinese Academy of Sciences, Beijing 100049, China; State Key Laboratory of Protein and Plant Gene Research, School of Advanced Agricultural Sciences, Peking University, Beijing 100871, China; Key Laboratory of RNA Biology, CAS Center for Excellence in Biomacromolecules, Institute of Biophysics, Chinese Academy of Sciences, Beijing 100101, China; University of Chinese Academy of Sciences, Beijing 100049, China; State Key Laboratory of Plant Genomics, Institute of Genetics and Developmental Biology, The Innovative Academy of Seed Design, Chinese Academy of Sciences, 100101 Beijing, China; State Key Laboratory of Plant Genomics, Institute of Genetics and Developmental Biology, The Innovative Academy of Seed Design, Chinese Academy of Sciences, 100101 Beijing, China; Key Laboratory of RNA Biology, CAS Center for Excellence in Biomacromolecules, Institute of Biophysics, Chinese Academy of Sciences, Beijing 100101, China; University of Chinese Academy of Sciences, Beijing 100049, China

## Abstract

Eukaryotic rRNAs and snRNAs are decorated with abundant 2′-*O*-methylated nucleotides (Nm) that are predominantly synthesized by box C/D snoRNA-guided enzymes. In the model plant *Arabidopsis thaliana*, C/D snoRNAs have been well categorized, but there is a lack of systematic mapping of Nm. Here, we applied RiboMeth-seq to profile Nm in cytoplasmic, chloroplast and mitochondrial rRNAs and snRNAs. We identified 111 Nm in cytoplasmic rRNAs and 19 Nm in snRNAs and assigned guide for majority of the detected sites using an updated snoRNA list. At least four sites are directed by guides with multiple specificities as shown in yeast. We found that C/D snoRNAs frequently form extra pairs with nearby sequences of methylation sites, potentially facilitating the substrate binding. Chloroplast and mitochondrial rRNAs contain five almost identical methylation sites, including two novel sites mediating ribosomal subunit joining. Deletion of FIB1 or FIB2 gene reduced the accumulation of C/D snoRNA and rRNA methylation with FIB1 playing a bigger role in methylation. Our data reveal the comprehensive 2′-*O*-methylation maps for Arabidopsis rRNAs and snRNAs and would facilitate study of their function and biosynthesis.

## INTRODUCTION

Most RNAs undergo posttranscriptional modifications that can play structural, functional and/or regulatory roles. More than 170 types of RNA modifications have been identified (1). In eukaryotes, 2′-*O*-methylated nucleotides (Nm) and pseudouridines (Ψ) are the two most frequent modifications in ribosomal RNAs (rRNAs). These modifications cluster on functionally important regions, such as peptidyl transferase center, decoding center and intersubunit interface, and are believed to fine tune the structure and function of ribosomes (2). Nm and pseudouridines in rRNAs are predominantly synthesized by box C/D and H/ACA small nucleolar ribonucleoproteins (snoRNPs), respectively, that rely on box C/D and H/ACA snoRNAs to recognize target sites by base-pairing interactions (3). In spliceosomal small nuclear RNAs (snRNAs), the two types of modifications are catalyzed by the related small Cajal body RNPs (scaRNPs) (4,5). A human tRNA was recently found to be 2′-*O*-methylated in an RNA-guided manner (6). These RNA-guided RNA modification enzymes are conserved in archaea where they modify rRNAs and tRNAs (7,8), but absent in bacteria where stand-alone protein enzymes synthesize a handful of Nm and pseudouridines in rRNAs (9).

C/D snoRNPs are composed of a distinct C/D snoRNA and four common proteins: the methyltransferase fibrillarin (FIB), the RNA-binding protein L7Ae and two paralogous scaffolding proteins NOP56 and NOP58 (10). C/D snoRNAs contain box C (RUGAUGA) and D (CUGA) motifs near the 5′ and 3′ ends, respectively, and the related box C' and D' motifs in the internal region. Box C and D and their nearby sequences fold into a kink-turn (K-turn) or K-loop structure that is characterized by tandem sheared GA pairs (11). Box C and D are essential for snoRNP assembly in cells, whereas box C' and D' are dispensable for snoRNP assembly and sometime degenerated. One or both spacer sequences linking two K-turns can make base pairing interactions with substrates and select a site that pairs to the fifth nucleotide upstream of box D' or D for modification (the D+5 rule) (12–14). Although C/D snoRNAs can display 10–20 nt of complementarity with substrates, substrates form a maximum of 10 base pairs (bp) with guides during modification in order to fit into the substrate-binding channel of C/D snoRNP (15–17). In the yeast *Saccharomyces cerevisiae*, C/D snoRNAs could form extra pairs in addition to primary pairs with substrates, aiding the modification (18). Other than functioning as methylation guide, a few special C/D snoRNAs, such as U3 and U14, are involved in rRNA processing and ribosome assembly (10). There are also orphan snoRNAs that have no known target in rRNA and snRNA.

Identification of all Nm in RNAs and assignment of responsible modification enzymes are fundamental for understanding their function and synthesis. In the well-studied budding yeast *Saccharomyces cerevisiae*, 55 Nm in rRNAs are synthesized by 44 C/D snoRNPs and a stand-alone protein enzyme Spb1 (G2922 of 25S) (19–22). Human rRNAs contain 112 Nm, most of which have been assigned with guide snoRNAs (23–27). Nm were traditionally identified individually (28). Recently, several approaches based on high-throughput sequencing were developed for systematic mapping of Nm (29–33). One of these methods, RiboMeth-seq, is based on the property that 2′-*O*-methylation prohibits alkali hydrolysis of phosphodiester bonds between modified nucleotides and their 3′ nucleotides (29). Methylation sites can be recognized by decrease of hydrolysis frequency of the RNA. The approach is also quantitative and capable of detecting small changes of methylation level (23,24,27,30,34,35).

In the model plant *Arabidopsis thaliana*, snoRNAs have been well characterized by computational prediction and verification of snoRNA genes and direct RNA sequencing (36–47). Plant snoRNA genes often have multiple variants and are arranged as polycistronic gene cluster (36–38,45,48–50). Multiple snoRNAs in cluster are transcribed into a precursor RNA that is processed into individual snoRNAs. In contrast to the wealthy information on snoRNAs, Nm have not been systemically mapped in Arabidopsis. The currently annotated Nm were largely predicted based on sequence complementarity to C/D snoRNAs and the D+5 targeting rule, and not all validated.

In this study, we have mapped Nm in cytoplasmic, chloroplast and mitochondrial rRNAs and snRNAs in Arabidopsis by RiboMeth-seq. We have assigned guide RNAs for majority of the detected Nm in cytoplasmic rRNAs and snRNAs and found evidence for atypical targeting and extra paring interactions with substrates in the action of Arabidopsis C/D snoRNAs. We further profiled Nm in fibrillarin and snoRNA mutant plants to validate some Nm and snoRNA assignments.

## MATERIALS AND METHODS

### Plant experiments

The ecotype of wild-type *Arabidopsis thaliana* was Columbia-0 (Col-0). The T-DNA insertion mutants CS858544 (*fib1-1*), SALK_093373C (*fib2-1*), SALK_134891 (U24.1), SALK_011503 (U24.2) and SALK_019614 (U24.2) were ordered from the Arabidopsis Biological Resource Center and SALK_047273C (SnoR58Y.2), SALK_117788C (SnoR29.2), SALK_092813C (SnoR10.1) and SALK_020249 (SnoR129) were ordered from the Nottinghan Arabidopsis Stock Centre. All mutants were verified by genotyping using the primers listed in Supplementary Table S1. The same *fib2-1* strain has been previously analyzed (51). SALK_017318 (*hid1*) and SALK_138192 (*hid2*) mutants were reported previously (52,53). Arabidopsis seed sterilization, stratification and standard seedling growth experiments were performed as previously described (53). Seedlings were light-grown for ∼9 days before isolation of total RNA with the Spectrum Plant Total RNA Kit (Sigma, STRN250).

### Detection of 2′-*O*-methylation by RiboMeth-seq

Sequencing libraries were prepared similarly as previously described (30). About 10 μg of total RNA was dissolved in 50 μl of 50 mM sodium carbonate/bicarbonate buffer (pH 9.2) and heated at 95°C for 10 min. Fragmented RNAs were diluted with 150 μl of diethyl pyrocarbonate treated water and precipitated by addition of 600 μl of ethanol, 20 μl of 3 M NaAc (pH 5.2) and 1 μl of 15 mg/ml GlycoBlue (Invitrogen). To prepare for ligation reaction, RNA was 3′-end dephosphorylated with 12.5 units of antarctic phosphatase (NEB) and purified with the RNA Clean & Concentrator-5 kit (Zymo Research). RNA was then 5′-end phosphorylated with 20 units of T4 polynucleotide kinase (NEB) and purified again as above.

Sequencing libraries were prepared with the NEBNext Small RNA Library Prep Set (NEB) following the manufacturer's instruction and using our own adaptors (Takara) and primers (Invitrogen) (Supplementary Table S2). An equal molar mixture of 5′ adaptor 1 and 2 were used. The 3′ adaptor was adenylated with the 5′ DNA adenylation kit (NEB). The 5′ and 3′ adaptors contain a 9- or 10-nt barcode composed of fixed and random sequences, which can be used to identify real RNA clones and remove PCR duplicates during data processing.

RNA was ligated to the 3′ and 5′ adaptors and reverse transcribed into cDNA. cDNA was PCR-amplified with 10–12 cycles using the P5 and P7 primers. Different samples were distinguished by the index sequences present in the P5 and P7 primers. PCR products were separated in 5% native PAGE gels and these in the range of 170–350 bp were excised. Excised gels were chopped into small pieces and socked in a buffer containing 10 mM Tris–HCl (pH 8.0), 300 mM NaCl and 1 mM EDTA for 13 h. DNA was precipitated by adding equal volume of isopropanol. Libraries with distinct indexes were mixed and sequenced with Illumina HiSeq X10 in the 150-bp paired-end mode by Annoroad Gene Technology. Each datasets contained 6–20 million reads.

### Data processing

The adaptor sequences were removed in Flexbar 3.1 with the options of adapter-error-rate = 0.2 and min-read-length = 33 (54). Barcodes and PCR duplicates were identified and removed with home-written scripts. Reads were aligned to the genome sequence of Arabidopsis (TAIR10, www.arabidopsis.org) and individual RNA sequences with HISAT2 using the no-spliced-alignment option (55).

The reference RNA sequences for mapping were derived from the genome sequence of Arabidopsis and revised, if needed, according to our sequencing data (Supplementary Table S3). Chloroplast and mitochondrial rRNAs also displayed single nucleotide polymorphism at several positions (Supplementary Table S3). The previous sequences of cytoplasmic 18S and 25S rRNAs (GeneBank ID: X16077 and X52320) differed in multiple places from their new sequences (Supplementary Table S4). Chloroplast 23S rRNA was processed into three fragments (Supplementary Figure S1) (56), to which reads were mapped separately.

Properly paired reads were extracted with SAMtools for further analysis (57). The number of 5′ and 3′ end of RNA fragments were counted from the 5′ end of aligned read 1 and 2, respectively, using bedtools genomecov (58). The 5′ end count was shifted one position upstream along the RNA sequence and combined with the 3′ end count. The methylation score (MethScore) was calculated as one minus the end count at a site divided by a weighted average of end counts at its neighboring sites, as described previously (29). Different from previous analyses, negative scores were not converted to zero. Twenty nucleotides at both ends of RNAs were not analyzed due to distorted end counts, but manually inspected for any possible modification. Read alignment and end coverage were viewed in IGV (59). Erroneous high score sites caused by low or uneven end coverage were manually removed. MethScores of cytoplasmic and chloroplast rRNAs were determined for all samples and listed in Supplementary Tables S4 and S5. MethScores of mitochondrial rRNAs and snRNAs were calculated from the pooled datasets from nine WT and *fib* samples and listed in Supplementary Tables S6 and S7.

### Compiling of snoRNAs

Box C/D and H/ACA snoRNAs are compiled from the SnoPY database, the Plant snoRNA database, the Araport11 annotation of Arabidopsis genome and original studies (36–39,41–47) (Supplementary Table S8). Chromosomal coordinates of snoRNAs were determined by alignment to the genome sequence of TAIR10 by BLASTN with parameters of evalue <1e−6 and similarity >95%. The names of several snoRNAs were revised according to the nomenclature convention (41). A previously found snoRNA, JKHR07A9 in SnoPY, was named as SnoR165, using the next consecutive number after the last named SnoR164 (43). The snoRNAs identified in our previous study were designated as SnoR143b, SnoR115.2, U27.1a, U27.1b, SnoR43.13 and SnoR166 to SnoR175 (45). U27.1a and U27.1b replaced the previous U27.1 snoRNA. Five novel snoRNAs identified in this study were designated SnoR176 to SnoR180 (Supplementary Table S9).

26 snoRNA genes were previously predicted based on promoter features (46). Twenty-four of these genes were not expressed in our sequencing data and the other ncR26 and ncR27 genes were expressed at low levels and with different transcript structures (Supplementary Table S10). These predicted snoRNAs were questionable and hence excluded from our analysis.

The abundance of snoRNAs was quantified relative to 18S rRNA, considering that cytoplasmic rRNAs have stable levels in different samples and were fully detected by RiboMeth-seq (Supplementary Table S8). The number of reads mapped to RNA was divided by the length of RNA in kilobases, yielding reads per kilobase (RPK). The RPK of each RNA was divided by that of 18S rRNA and multiplied by 1 million, yielding the normalized transcripts per million (TPM). The normalized TPM strands for the number of RNA molecules for every million of 18S rRNA molecules and allows comparison of RNA abundance in different samples.

### Searching base pairing interactions between methylation sites and C/D snoRNAs

Sequences of 100 nt flanking each methylation site were extracted and aligned to all C/D snoRNAs by BLASTN with the option of ‘-task ‘blastn-short’ -evalue 100 -strand minus’ (60). Interactions as short as seven Watson-Crick pairs can be identified in this way. The primary pair must contain at least 2- and 3-bp on the 5′ and 3′ side of methylation site in substrate, respectively. The methylation site must pair to the fifth nucleotide upstream of box D/D' with the ‘NNGA’ sequences. Extra pairs that overlapped significantly with box C/D motifs or major pairs were not considered. As GU wobble pair was treated as mismatch in BLASTN, the identified base-pairing interactions were further extended at both sides by allowing GU pair. The interaction between Nm and C/D snoRNA was also analyzed with Snoscan (22). Compared to the BLASTN search, Snoscan was more tolerated to mismatch in pairing and mutation in box D'/D and preferred longer pairing interactions. Several hits found by Snoscan were adopted where the BLASTN search identified no guide. The guide RNAs assigned for cytoplasmic rRNAs and snRNAs are listed in Supplementary Table S11 and S12, respectively.

## RESULTS

### RiboMeth-seq mapping of Nm

To map Nm in Arabidopsis RNAs, total RNAs were extracted and hydrolyzed briefly at mild alkaline conditions. The fragmented RNAs were converted into cDNA libraries that were sequenced to produce 150-bp paired-end reads. The reads were mapped to the Arabidopsis genome sequence and individual reference RNA sequences that were sometime revised according to our sequencing data (Supplementary Table S3). The 5′ and 3′ end of RNA fragments were counted for each position and combined to calculate the MethScore (Supplementary Table S4-S7) (29). MethScore has a maximum of one when a site is fully methylated and not hydrolyzed, and is reduced when a site is partially methylated and undergoes some degree of hydrolysis.

### Nm in cytoplasmic rRNAs

**Figure 1.**
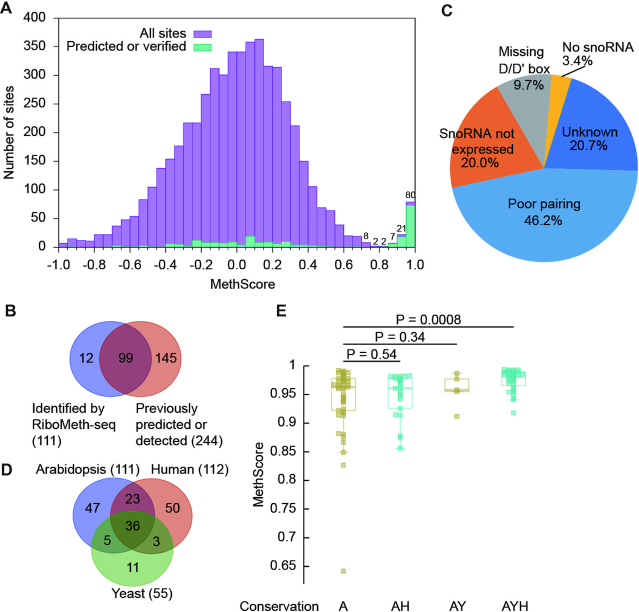
RiboMeth-seq analysis of Arabidopsis cytoplasmic rRNAs. (**A**) Histogram of methylation scores (MethScore) for all analyzed sites in cytoplasmic rRNAs. Means of *n* = 3 biological replicates were counted. Sites with MethScore < -1 are not shown. Numbers of sites are labeled for the top bins. The upper boundary of each bin is inclusive and the lower boundary is not. (**B**) Venn diagram comparing the Nm identified by RiboMeth-seq and the previously predicted or experimentally detected methylation sites. (**C**) Pie chart showing possible reasons for 145 not confirmed methylation sites. (**D**) Venn diagram showing conservation of Nm among Arabidopsis, S. cerevisiae and human rRNAs. (**E**) Box plots of MethScore for Nm with different conservation. A: Arabidopsis, Y: yeast, H: human. *P* values are from two-tailed *t*-test.

MethScores of cytoplasmic rRNAs were well separated into a high and low score group (Figure 1A). Majority of the high score sites corresponded to the previously predicted targets of C/D snoRNAs or experimentally detected sites, validating the specificity of our data (Supplementary Table S11). The low score group should contain the vast unmethylated sites and potentially some sites with low degree of methylation. MethScores were widely distributed in the low score group, suggesting that the intrinsic hydrolysis rate varies greatly for unmethylated sites. Using a threshold of 0.8, 110 sites were identified as Nm. The threshold was chosen so that the closest site above the threshold had predicted guide RNAs, whereas the closest site below the threshold did not.

Partially methylated nucleotides with MethScores as low as ∼0.5 have been detected (20,24,25,29,30). Given the wide distribution of MethScores for unmethylated sites, assignment of low score methylation sites needed additional evidence, such as presence of guide RNA and sensitivity to *fib* mutants. Among all sites with MethScore between 0.5 and 0.8, only 25S_U803 and 18S_U1107 had a predicted guide RNA (Supplementary Table S4). U803 made a strong interaction with SnoR30 and showed significantly reduced methylation in the *fib* mutants (see below). By contrast, the methylation level of U1107 was not affected in the *fib* mutants. U1107 was predicted to be targeted by SnoR35, but the predicted interaction contained one mismatch and SnoR35 was not expressed (Supplementary Table S8). Hence, only 25S_U803 was assigned as low score methylation site.

Together, we identified 111 Nm with high confidence, including 35 in 18S, 74 in 25S and 2 in 5.8S (Table 1 and Supplementary Table S11). No methylation site was found in 5S rRNA. 99 sites were previously predicted and/or experimentally identified and 12 sites were novel (Figure 1B). 145/244 of the previously predicted and/or experimentally identified sites were not confirmed by RiboMeth-seq (Figure 1B). Most of these not confirmed sites were predicted on weak guide-target interactions (46.2%), unexpressed snoRNAs (20%) or guides that miss a box D/D' motif (9.7%) (Figure 1C). Some may be methylated in low degrees and escaped the RiboMeth-seq detection. Thus, we have obtained a highly accurate and complete map of Nm in cytoplasmic rRNAs. The large difference between our and the previously predicted Nm maps also necessitates experimental identification of Nm.

**Table 1.** Nm in Arabidopsis cytoplasmic rRNAs and assigned C/D guide snoRNAs

*18S rRNA*		*25S rRNA*			
A28	^a^U27	U44	SnoR120	G2289	^a^U15
C38	SnoR66	U48	^a^SnoR16	C2294	SnoR131-133
U123	SnoR116	U144	SnoR36	A2322	U30
A162	^a^SnoR18	G399	SnoR65	A2327	^a^SnoR44
U213	SnoR65, SnoR146	A661	^a^U18	C2338	^a^SnoR44
G246	SnoR124	C675	^a^SnoR58Y	A2362	SnoR43.12
G392	SnoR30	U676	^c^SnoR58Y	C2366	^a^SnoR37
C418	^a^U14	U803	^a^SnoR14	G2392	^a^SnoR29
A440	SnoR15,U16	G814	^a^SnoR39BY	G2396	^a^SnoR28
A468	SnoR17	A816	^a^U51	G2410	^a^SnoR29
C473	^a^SnoR7	A826	^a^U80	U2411	^c^SnoR29
A545	SnoR41Y	A885	^a^SnoR72Y	U2422	^a^SnoR37
U582	^a^SnoR77Y	G917	^a^U80	U2456	SnoR16.1
G599	U54	A945	^a^SnoR12	U2494	^a^SnoR123
U604	^a^SnoR115	U1067	SnoR41Y	G2620	SnoR35, ^a^U31
U615	^a^SnoR13	A1143	^a^U38	A2641	SnoR27, SnoR68Y
A623	U36	A1263	^a^SnoR22	U2651	^a^SnoR10
A780	SnoR119	U1278	^a^SnoR22	G2652	^c^SnoR10
A796	SnoR25	A1377	^a^SnoR7	C2683	SnoR148
A801	SnoR53Y	C1447	^a^U24	U2736	SnoR68
A978	^a^SnoR59	A1459	^a^U24	G2792	^a^SnoR1
U1013	SnoR20.1	G1460	^c^U24	G2794	^d^SnoR1
C1219	SnoR166	C1479	SnoR147	G2816	^a^SnoR38Y
U1235	^a^SnoR14	C1518	^a^U49, SnoR121	C2837	^a^SnoR24
U1264	^a^SnoR8, SnoR67	C1847	SnoR128-129	C2880	^a^U49
U1266	^a^SnoR32	C1850	SnoR149	U2884	SnoR64
U1273	^a^U33, SnoR34	G1855	^a^SnoR59	A2912	SnoR31
G1275	^a^SnoR21	C1860	SnoR15,U55	G2918	SnoR34, SnoR6.3
A1330	^a^SnoR32	U1892	^a^U34	U2922	
U1384	U61.1	U2114	SnoR117	G2923	By Spb1 in yeast
G1434	^a^SnoR19	G2125	^a^U60	A2935	^a^SnoR18
U1448	^a^SnoR19	A2127	^a^SnoR12	A2947	U29
A1579	^a^SnoR8	C2198	SnoR118	C2949	SnoR69Y
C1645	^a^U43	A2215	U37	C2960	U35
A1758	^a^SnoR23	A2221	^a^U36	G3292	^a^U33
*5.8S rRNA*		G2237	^a^U36	U3301	^a^SnoR13
A47	^a^SnoR9	A2257	^a^U40		
G79	^a^SnoR39BY	A2282	^a^U15		

^a^Multiple gene variants are not shown.

^b^Underlined are the novel methylation sites that were not previously predicted or detected.

^c^Validated non-canonical targeting.

^d^Predicted non-canonical targeting.

36/111 methylation sites are conserved in yeast and humans (Figure 1D, Supplementary Table S13), and they showed significantly higher levels of methylation than those present only in Arabidopsis (Figure 1E). These highly conserved methylation sites represent the core set of Nm in cytoplasmic rRNAs and tend to be fully methylated. Those methylation sites conserved only in humans or yeast did not show significant difference in methylation level compared to Arabidopsis-specific sites.

### Target recognition by C/D snoRNA

We have compiled a comprehensive list of snoRNA (Supplementary Table S8), which contains one C/D and four H/ACA novel snoRNAs identified based on our sequencing data (Supplementary Table S9). Several snoRNAs were not expressed, including those predicted based on promoter features (Supplementary Table S10) (46). In total, C/D snoRNAs have 118 different genes and 214 gene variants and H/ACA snoRNAs have 72 different genes and 104 gene variants. If excluding the genes with no expression, there are 108 different genes and 184 gene variants for C/D snoRNAs and 69 different genes and 96 gene variants for H/ACA snoRNAs.

To assign guide RNA for the identified Nm, the sequence complementarity between 100 nucleotides flanking each Nm site and all C/D snoRNAs was examined by a BLASTN-based approach (See Materials and Methods). The approach allowed to identify primary pairs between methylation sites and guide RNAs, as well as extra pairs that potentially strengthened the target recognition (18).

**Figure 2.**
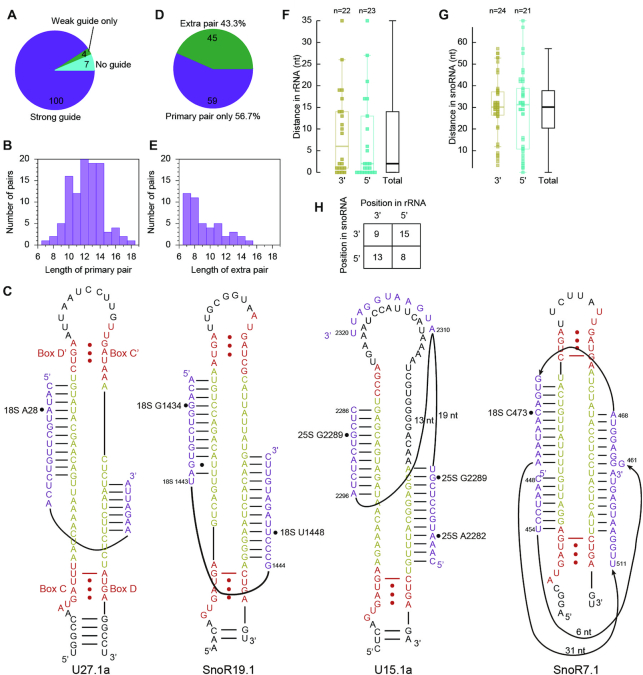
Base pairing interactions between C/D snoRNAs and detected Nm in cytoplasmic rRNAs. (**A**) Pie plot showing guide RNA assignment for detected Nm. (**B**) Histogram of length of primary pairs. (**C**) Examples of extra pairing between C/D snoRNAs and target sites. Box C/D and C'/D' are colored in red, spacers yellow and substrates purple. Methylation sites are marked by circles. Box C' is not recognized in U15.1a. (**D**) Pie plot showing occurrence of extra pairing. (**E**) Histogram of length of extra pairs. (**F, G**) Box plots showing the distance between primary and extra pairs as measured from rRNA (F) and C/D snoRNA (G). Extra pairs are classified according to their location at the 3′ or 5′ side of major pairs or shown together (Total). (**H**) Statistics on the relative position of extra pairs to major pairs as seen from rRNAs and snoRNAs.

Guide RNAs were assigned for 104/111 Nm following the D+5 rule, including all sites in 18S and 5.8S rRNAs (Figure 2A, Table 1 and Supplementary Table S11). Seven sites in 25S (U676, G1460, U2411, G2652, G2794, U2922 and G2923) remained unassigned. 9/104 assigned sites are targeted by two and more different C/D snoRNAs (not counting variants). The predicted guide-target interactions for 100/104 Nm are composed of at least nine consecutive base pairs that could contain GU wobble pairs at terminal regions, but exclude any mismatch (Figure 2A). Most of the guide-target interactions comprise of 10–14 bp (Figure 2B). The extent of pairing between snoRNAs and experimentally detected Nm suggests that strict criteria should be generally applied in target prediction of C/D snoRNA.

The predicted interactions of SnoR1a/25S_G2792 and U36a/25S_A2221 start at position 4 of the guide (counted from box D/D'). In the interactions of SnoR25/18S_A796, SnoR32/18S_A1330 and SnoR29.2/25S_G2410, the guide is adjacent to a degenerated box D'. The predicted interactions for four sites were weak and less certain. SnoR129 and related SnoR128 potentially form a 14- and 13-bp duplex with the target sequence of 25S_C1847, but A1851 needs to be bulged out (Supplementary Figure S2A). We have validated that 25S_C1847 is indeed targeted by SnoR129 (Supplementary Figure S2B-D). The SnoR69Y/25S_C2949 and SnoR43.12/25S_A2362 interactions involve 8-bp duplexes, but the former interaction is conserved in yeast. Finally, the SnoR28/25S_G2396 interaction contains a 7-bp major pair and an 8-bp extra pair.

45/104 Nm were found to make extra 7 to 15 bp interactions with at least one of their assigned guides (Figure 2C–E, Supplementary Table S11). More than half of extra pairs are immediately adjacent to primary pairs in substrate (Figure 2F), suggesting that they are not formed randomly and hence relevant. Primary and extra pairs frequently occur at different spacers with a median distance of 30 nt in snoRNA (Figure 2G). Last, primary and extra pairs show no preference (*P* value > 0.5, Chi-square test) in terms of their relative position in either substrate or snoRNA (Figure 2H).

As special cases of extra pairing, six snoRNAs (SnoR19, SnoR22, U24, U36, SnoR44 and SnoR29) employ their dual antisense elements to target two sites separated by 11–18 nt (Table 1, Figure 2C). In principle, the two target sites can bind simultaneously to the snoRNAs, with each forming an extra pair for the other. Another special example is U15 that targets A2282 and G2289 of 25S rRNA with its D and D' guide, respectively. The two target sites are too close to bind two spacers of U15 simultaneously (Figure 2C). Their binding is potentially aided by a common extra pair formed on the top loop of U15. Most extra pairs occur singly, but SnoR7 and U49 seem to form three extra pairs with their targets (Figure 2C, Supplementary Table S11).

Among the seven Nm with unassigned guide, the consecutive nucleotides U2922 and G2923 in 25S rRNA are located at the A-loop and highly conserved in all types of rRNAs (Table 2). The equivalent of G2923 in yeast is modified by a standalone protein enzyme Spb1 (21). The Arabidopsis orthologue of Spb1 (AT4G25730) most likely catalyzes the same modification. Methylation of the equivalent of U2922 is guided by the snR52 snoRNA in yeast, but a reliable guide cannot be identified in Arabidopsis. In theory, U2922 could be modified by an unknown C/D snoRNP as in yeast or a protein enzyme as in *E. coli* and mitochondria.

**Table 2.** Nm in Arabidopsis chloroplast and mitochondrial rRNAs and their equivalents in other rRNAs

Arabidopsis Pt	Arabidopsis Mt	Arabidopsis cytoplasm	Yeast cytoplasm	Human cytoplasm	^a^Yeast Mt	^a^Human Mt	^b^ *E. coli*	Location
SSU								
Cm1351	Cm1751	Cm1645	Cm1639	Cm1703			m^4^Cm1402	Decoding center
Cm1358	Cm1758						C1409	Subunit interface
LSU								
Cm1935	Cm2216						C1920	Subunit interface
Gm2269	Gm2538	Gm2620	Gm2619	Gm4166	Gm2270	Gm1145	Gm2251	P-site
	Um2835	Um2922	Um2921	Um4468	Um2791	Um1369	Um2552	A-site
Gm2571		Gm2923	Gm2922	Gm4469		Gm1370	G2553	A-site

^a^Nm in yeast and human mitochondrial SSU rRNAs are not available.

^b^The equivalent nucleotides in *E. coli* rRNA are listed.

### Guide RNAs with multiple specificities

Classic C/D snoRNAs select a site paired to the fifth position upstream of box D/D' for modification. This rule is the basis for target prediction of C/D snoRNAs. However, four yeast snoRNAs (U18, snR13, U24 and snR48) have been found to guide modification of two close sites using a single guide sequence upstream of box D' (13,22,61), breaking the consensus rule of targeting. The two target sites are consecutive at positions 5 and 6 upstream of box D' for yeast U18, snR13 and U24 and separated by one nucleotide for snR48. Based on the conservation with yeast in position of Nm and pattern of snoRNA-rRNA interaction, we suggest that five of the unassigned Nm in Arabidopsis rRNAs may be targeted by snoRNAs with multiple specificities.

**Figure 3.**
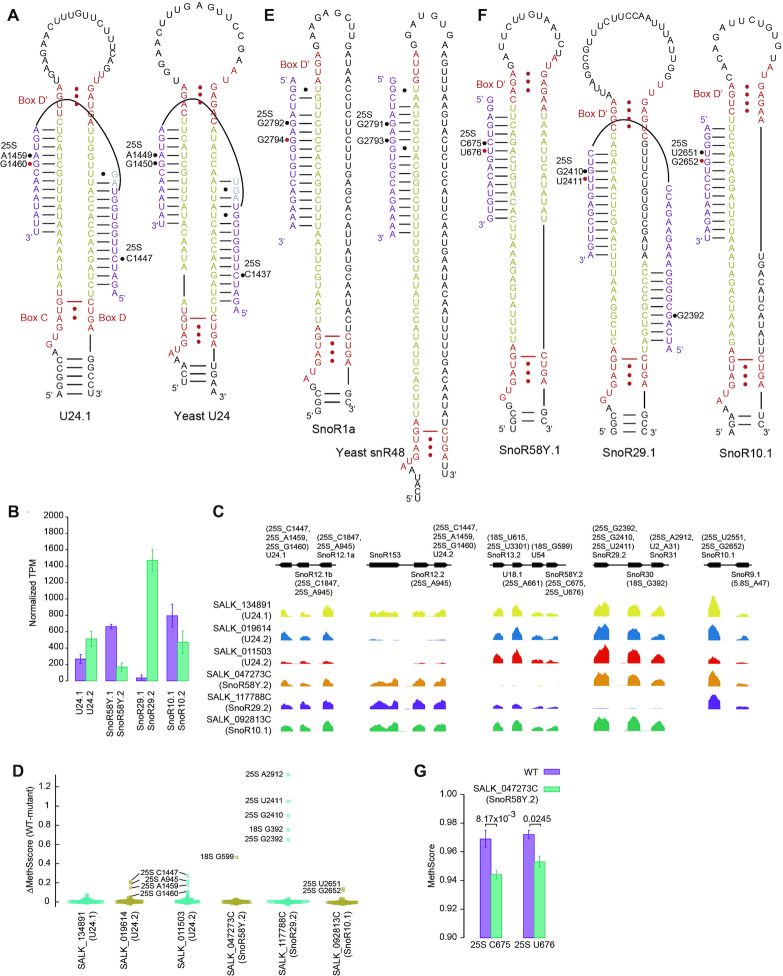
Arabidopsis C/D snoRNAs with multiple specificities. (**A**) Comparison of Arabidopsis and yeast U24. Box C/D and C'/D' are colored in red and spacers are yellow. Substrate rRNA is purple and the nucleotides involved in competing interactions are grey. The Nm with or without assigned guide are marked with black or red circles. (**B**) Expression levels of snoRNA variants in the WT plant. Mean and SD were calculated from *n* = 3 samples. (**C**) Read coverage of snoRNAs in the analyzed T-DNA mutants normalized against 18S rRNA. The gene organization of snoRNA clusters and the predicted targets of C/D snoRNAs are displayed. (**D**) Beeswarm plot showing MethScore changes between WT and mutant plants for all identified Nm in cytoplasmic rRNAs. The sites with prominent reduction of methylation are labeled. MethScores were calculated as means of *n* = 3 independent samples for wild-type, *n* = 2 for SnoR58Y.2, SnoR29.2 and SnoR10.1 mutants and *n* = 1 for three U24 mutants. (**E**) Comparison of Arabidopsis SnoR1 and yeast snR48. Box C' is not assigned. (**F**) Three additional C/D snoRNAs with multiple specificities. (**G**) MethScores of two target sites of SnoR58Y. Mean and SD were calculated from *n* = 3 WT samples and *n* = 2 SnoR58Y.2 mutant samples. *P* values are from two-tailed *t*-test.

The yeast U24 snoRNA directs the methylation of C1437 of 25S by the D guide and A1449 and G1450 of 25S by the D' guide (Figure 3A). The three equivalent sites (C1447, A1459 and G1460) in Arabidopsis are all methylated. The first two sites are guided by U24 according to the D+5 rule and the last one is unassigned. Given the conservation of the three methylation sites and U24 snoRNA in yeast and Arabidopsis, G1460 is probably also targeted by the D' guide of U24. To validate the prediction, we analyzed rRNA methylation in three T-DNA mutants of U24 that have two variants expressed at different levels (Figure 3B and C). Indeed, two mutants (SALK_019614 and SALK_011503) that blocked the expression of the more abundant U24.2 variant reduced the methylation of all three predicted target sites of U24 (Figure 3D). The expression of SnoR12.2 in the U24.2 cluster and methylation of its predicted target 25S A945 were also affected. A mutant of U24.1 (SALK_134891) was ineffective as neither the expression level of U24.1 nor the methylation of its targets were reduced (Figure 3C and D).

Yeast snR48 guides the methylation of G2791 and G2793 of 25S (Figure 3E). The equivalent positions G2792 and G2794 in Arabidopsis rRNAs are both methylated. G2792 is targeted by SnoR1 and G2794 remains unassigned. Following the same reasoning for U24, G2794 may be targeted by the D' guide of SnoR1, which appears to be the orthologue of yeast snR48. The prediction has not been validated as no suitable T-DNA mutant could be found for two SnoR1 variants. Multiple specificities of yeast U18 and snR13 are not conserved in Arabidopsis; their target sites at position 5 are methylated in Arabidopsis but those at position 6 are not (Supplementary Table S11).

Moreover, three unassigned Nm, U676, U2411 and G2652 of 25S, are all located downstream of a methylated nucleotide (C675, G2410 and U2651) that is targeted by the D' guide of a C/D snoRNA (SnoR58Y, SnoR29 and SnoR10) (Figure 3F). The pattern of tandem Nm and targeting by D' guide are reminiscent of multiple specificities of yeast U24, U18 and snR13. We speculate that these three sites may be guided by snoRNAs that target their 5′ sites. If true, SnoR29 would guide the methylation of three sites (G2392, G2410 and U2411 of 25S) in a similar way as U24. These snoRNAs each have two variants (Figure 3B). Blocking the expression of the more abundant SnoR29.2 and SnoR10.1 variants significantly decreased the methylation of their canonical and non-canonical targets (Figure 3C and D). In a T-DNA mutant that eliminated the less abundant SnoR58Y.2 variant (Figure 3C), the methylation of C675 and U676 was slightly, yet statistically significantly, reduced (Figure 3G). Therefore, we conclude that U676, U2411 and G2652 of 25S are all selected by the non-canonical targeting rule.

### Nm in chloroplast and mitochondrial rRNAs

**Figure 4.**
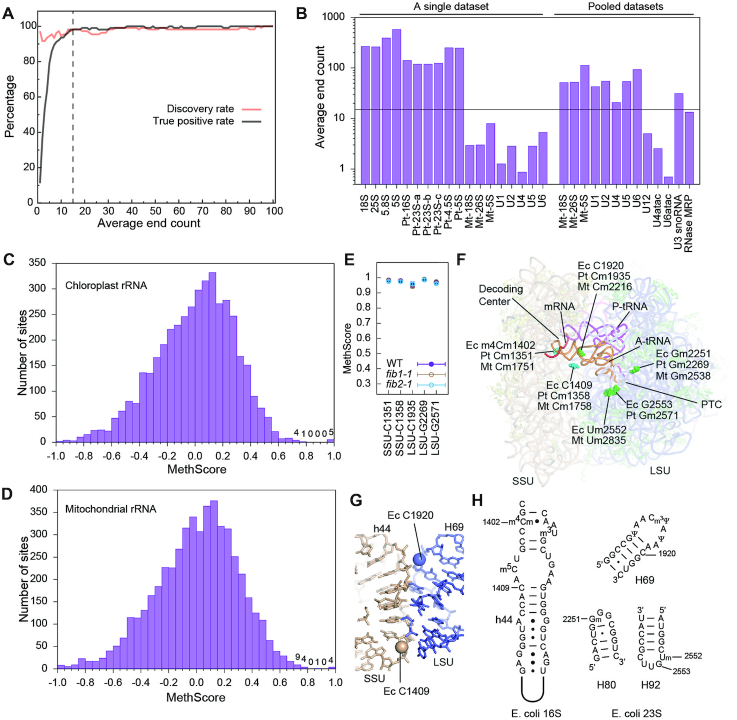
Nm in Arabidopsis chloroplast and mitochondrial rRNAs. (**A**) Plot of discovery rate (True Positive/True) and true positive rate (True Positive/Positive) as a function of average end count (AEC). The sequencing dataset was reduced in size and used to calculate MethScores for 18S and 25S rRNAs. Sites with MethScore >0.8 are called positive. The reference true set consists of all identified Nm but 25S_U803 and the 5.8S sites. The dashed line marks AEC = 15. (**B**) Bar plot showing AEC for several abundant RNAs in a single RiboMeth-seq dataset (WT_rep1, 12.7 M reads) and the pooled datasets (131 M reads). The minimal level of AEC = 15 required for reliable methylation identification is shown. Pt: chloroplast, Mt: mitochondrial. (**C, D**) Histogram of MethScores for chloroplast rRNAs (mean of *n* = 3 biological replicates) (C) and mitochondrial rRNAs from the pooled datasets (D). The sites with score <–1 are not shown. Numbers of sites are labeled for the top bins. (**E**) MethScores are plotted as mean ± SD (*n* = 3) for the detected Nm in chloroplast rRNAs of WT and *fib* mutant plants. (**F**) The Nm in Arabidopsis chloroplast and mitochondrial rRNAs mapped to the structure of Thermus thermophilus 70S ribosome bound with mRNA and tRNAs (PDB code: 4V5D). Nm are shown as spheres and colored in cyan for the SSU and green for the LSU. The equivalent residues in E. coli (Ec), Arabidopsis chloroplast (Pt) and mitochondrial (Mt) rRNAs are labeled for each methylation site. PTC, peptidyl transferase center. (**G**) A zoom-in view of the subunit interface. The 2′-hydroxyl groups of the novel methylation sites are shown as spheres. (H) Secondary structures and modifications in *E. coli* rRNAs.

Calculation of reliable MethScores critically depends on adequate read coverage or more directly on end counts. We assessed the average end count (AEC), which equals to the total number of reads (one read in a pair) mapped to RNA divided by the length of RNA, as an indicator of overall MethScore reliability. To estimate the minimal level of AEC required for reliable identification of Nm, we conducted a simulation experiment where MethScores were determined on gradually reduced numbers of reads (Figure 4A). The discovery rate was relatively insensitive to the AEC, but the true positive rate abruptly dropped when the AEC was <15. As a rule of thumb, the AEC should be >15 for reliable identification of Nm.

The AEC is shown for several abundant RNAs in a typical RiboMeth-seq experiment (Figure 4B). Cytoplasmic and chloroplast rRNAs are the most abundant RNA species with AEC in order of 100 and can be analyzed reliably (Figure 4C). By contrast, mitochondrial rRNAs are two orders of magnitude less abundant than the other two rRNAs, which prevents reliable determination of MethScore. To increase the read coverage, nine datasets from wild-type and *fib* mutant plants were pooled, which raised the AEC to a level that Nm can be reliably identified for mitochondrial rRNAs and snRNAs (Figure 4B, D, Tables 2 and 3, Supplementary Tables S6-S7).

**Table 3.** Nm in Arabidopsis snRNAs and assigned C/D guide snoRNAs

*U2 snRNA*		*U4 snRNA*		*U6 snRNA*	
G13	SnoR113	A67		U27	SnoR167
G20	SnoR127	*U5 snRNA*		A43	^a^U27
G26		G40	^a^SnoR102	A48	SnoR53Y
C29	SnoR101	U44		C57	
A31	SnoR125, SnoR31	C48	SnoR130	C63	SnoR26
A39	^a^SnoR24			A65	
C41	SnoR176			G75	SnoR126
				G84	SnoR126

^a^Multiple gene variants are not shown.

^b^Underlined are the novel methylation sites that were not previously predicted or detected.

Using a threshold of 0.8, five Nm were identified with high confidence for chloroplast rRNAs, including Cm1351 and Cm1358 in the small subunit (SSU) rRNA and Cm1935, Gm2269 and Gm2571 in the large subunit (LSU) rRNA (Figure 4C, E–H, Table 2). The MethScores of these sites were all unaffected in the *fib* mutants, suggesting that they are synthesized by stand-alone protein enzymes rather than C/D snoRNPs (Figure 4E). Cm1351 of the SSU rRNA is located in the decoding center and also conserved in cytoplasmic rRNAs. The corresponding nucleotide in *E. coli* is a N^4^, 2′-O-dimethylcytidine (m^4^Cm1402). Gm2269 and Gm2571 of the LSU rRNA are located at the peptidyl (P)- and aminoacyl(A)-site of the peptidyl transferase center (PTC), respectively, and highly conserved in all types of rRNA. Of note, Cm1358 of the SSU rRNA and Cm1935 of the LSU rRNA have not been found to be modified in other rRNAs (Table 2). These two sites are located at the intersubunit interface and their methyl groups would directly mediate the packing between helix 44 (h44) of SSU and helix 69 (H69) of LSU (Figure 4G), suggesting that methylation at the two sites regulates the joining of ribosomal subunits.

MethScores were calculated from the pooled datasets for mitochondrial rRNAs (Figure 4D). Due to uneven end coverage, several sites with artificial high scores needed to be removed (Supplementary Figure S3A). Five Nm were identified using a threshold of 0.8, including Cm1751 and Cm1758 in the SSU rRNA, and Cm2216, Gm2538 and Um2835 in the LSU rRNA (Table 2, Figure 4F). Remarkably, the first four sites are identical to those in chloroplast rRNAs and Um2835 is adjacent to the equivalent of Gm2571 of chloroplast rRNAs. The equivalent nucleotide of Um2835 is also methylated in yeast and human mitochondrial rRNAs (62–64) and *E. coli* rRNA. The A-loop contains a UG dinucleotide that is 2′-O-methylated at one or both of sites (Table 2). Both sites are methylated in eukaryotic cytoplasmic rRNAs and only one and different site is modified in Arabidopsis chloroplast and mitochondrial rRNAs. Arabidopsis chloroplast rRNAs represent a unique case where the G is modified and the U is unmodified.

### Nm in snRNAs and other RNAs

**Figure 5.**
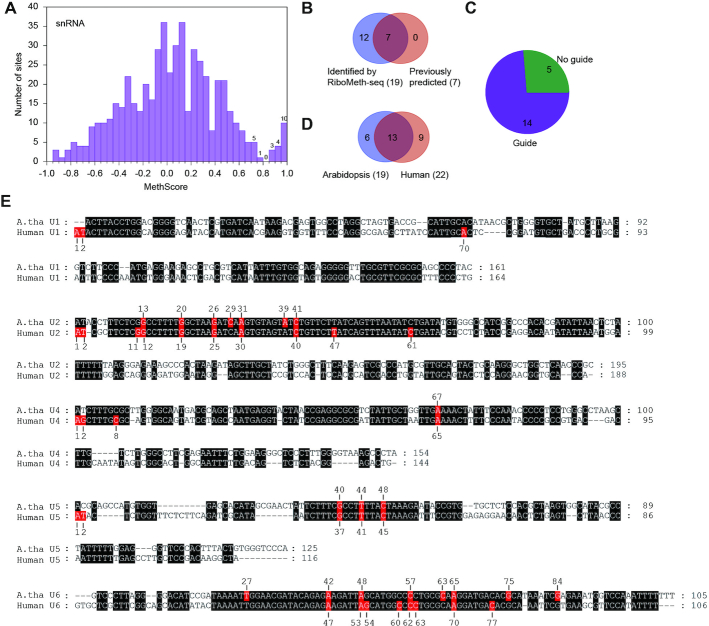
Nm in Arabidopsis snRNAs. (**A**) Histogram of MethScores for all analyzed sites in five snRNAs. The data were based on the pooled datasets and do not include twenty residues at the 5′ and 3′ termini of snRNAs. Gm13 and Gm20 in U2 snRNA were manually identified. The sites with MethScore < -1 are not shown. Numbers of sites are labeled for the top bins. (**B**) Venn diagram comparing the Nm identified by RiboMeth-seq and the previously predicted methylation sites. (**C**) Pie plot showing guide RNA assignment for the detected Nm in snRNAs. (**D**) Venn diagram showing conservation of Nm between Arabidopsis and human snRNAs. The terminal Nm in human snRNAs are not counted. (**E**) Alignment of Arabidopsis thaliana (A. tha) and human snRNAs. Identical residues are shaded in black. Nm are shaded in red and labeled.

A total of 19 Nm was identified in snRNAs with the pooled datasets, including zero in U1, seven in U2, one in U4, three in U5 and eight in U6 (Table 3, Supplementary Table S7 and S12, Figure 5A and Supplementary Figure S4). All previously predicted seven sites were detected and 12 sites were novel (Figure 5B). 14/19 sites have been assigned with guide snoRNAs and six sites form extra pairing interactions with the snoRNAs (Table 3 and Supplementary Table S12, Figure 5C). These predicted guide snoRNAs are presumably located in the Cajal body as human scaRNAs, but their localization and mechanism of localization have not been characterized (65,66). 13/19 sites are also methylated in human snRNAs (4,67), underscoring their important function (Figure 5D-E). These Nm are located in highly conserved, functionally important regions of snRNAs that are involved in RNA-RNA interaction and catalysis of pre-mRNA splicing. In human U1, U2, U4 and U5, the first and second nucleotides are 2′-*O*-methylated by CMTr1 and CMTr2 during the formation of the cap structure (68). The two positions were not methylated in Arabidopsis snRNAs (Supplementary Figure S4).

The AEC of minor spliceosomal snRNAs was insufficient for Nm determination (Figure 4B). The U3 snoRNA and RNase MRP RNA that are involved in rRNA processing contained no Nm, although they show adequate end coverage in the pooled datasets (Figure 4B, Supplementary Figure S3B and C).

### SnoRNA cluster mutants

**Figure 6.**
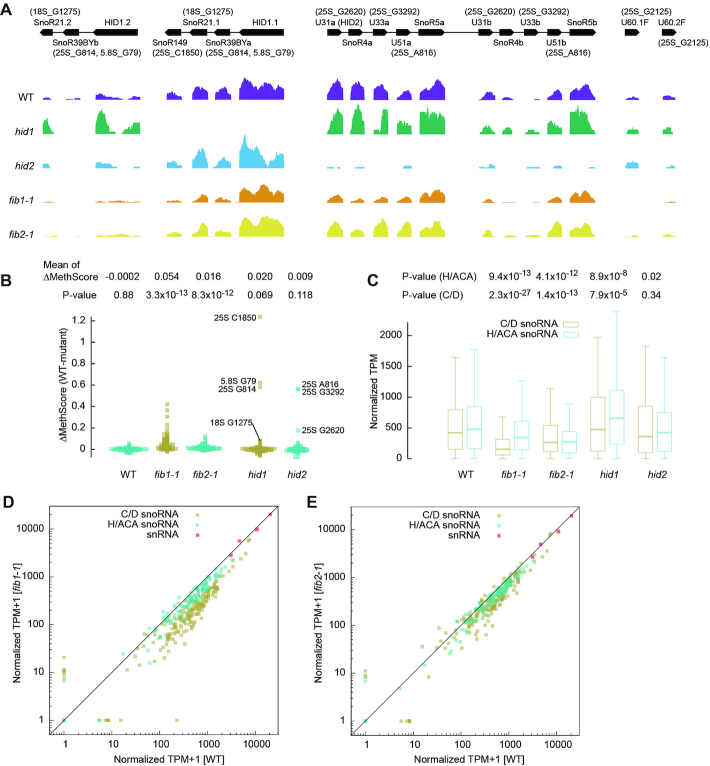
rRNA 2′-O-methylation in snoRNA and FIB mutants. (**A**) Read coverage normalized against 18S rRNA is shown for the HID1 and HID2 snoRNA clusters, U60.1F (FIB1 intron) and U60.2F (FIB2 intron) in wild-type and mutant plants. The gene organization of snoRNA clusters and the predicted targets of C/D snoRNAs are displayed. (**B**) Beeswarm plot showing MethScore changes between WT and mutant plants for all identified Nm in cytoplasmic rRNAs. The targets of C/D snoRNAs affected in *hid1* and *hid2* are labeled. Means of MethScore changes and p-values from one-tailed paired t-test are shown. MethScores are means of *n* = 3 independent samples for wild-type, *fib1-1* and *fib2-*1 and from one measurement for *hid1* and *hid2*. The WT group was calculated between two independent wild-type samples. (**C**) Box plot showing abundance of C/D and H/ACA snoRNAs. Transcripts per million (TPM) were normalized against 18S rRNA (as 1 M). The central line indicates median value, while the box and whiskers represent the interquartile range (IQR) and 1.5 × IQR, respectively. *P* values are from two-tailed paired *t*-test. (**D, E**) Abundance of C/D snoRNAs, H/ACA snoRNAs and snRNAs in *fib1-1* (D) and *fib2-1* (E) is compared to that in wild-type. RNA abundance data shown in C-E are means of *n* = 3 independent samples for wild-type, *fib1-1* and *fib2-*1 and from one measurement for *hid1* and *hid2*.

To validate some of the identified Nm and their assigned guide RNAs, we profiled Nm in two snoRNA cluster mutants *hid1* and *hid2* analyzed previously (52,53). *hid1* was a T-DNA insertion mutant that disrupted the expression of a polycistronic snoRNA cluster composed of a HID1 ncRNA and three C/D snoRNAs SnoR39BYa, SnoR21.1 and SnoR149 (Figure 6A, Supplementary Table S8) (53). The targets of SnoR39BYa (25S_G814 and 5.8S_G79) and SnoR149 (25S_C1850) showed greatly decreased methylation in *hid1*, but the target 18S_C1275 of SnoR21.1 was only slightly reduced in methylation (Figure 6B). The methylation of 18S_C1275 was also guided by the SnoR21.2 variant whose expression was rather increased in *hid1* (Figure 6A, Supplementary Table S8), accounting for the observation.

*hid2* was a T-DNA insertion mutant that disrupted the expression of two duplicated snoRNA clusters (Figure 6A, Supplementary Table S8) (52). In the clusters, SnoR4a/b are orphan C/D snoRNAs without a known target and SnoR5a/b are H/ACA snoRNAs. U31a/b (HID2), U33a/b and U51a/b were predicted to guide the methylation of 25S_G2620, 25S_G3292 and 25S_A816, respectively. All three sites were significantly decreased in methylation in *hid2* (Figure 6B). Our data confirm the predicted targets for the three C/D snoRNAs and no target in rRNAs for SnoR4. 25S_G2620 was still modified to a considerable level (MethScore = 0.816) in *hid2* probably because U31a and U31b retained 27% of their original expression levels (Figure 6A, Supplementary Table S11). The change of 25S_G2620 methylation was not previously detected by conventional methods that are less sensitive than RiboMeth-seq (52).

The expression levels of C/D and H/ACA snoRNAs were unchanged in *hid2*, but increased by 24% and 31%, respectively, in *hid1* (Figure 6C, Supplementary Table S8). This may be related to the defective photomorphogenesis caused by *hid1* (53).

### Fibrillarin mutants

**Figure 7.**
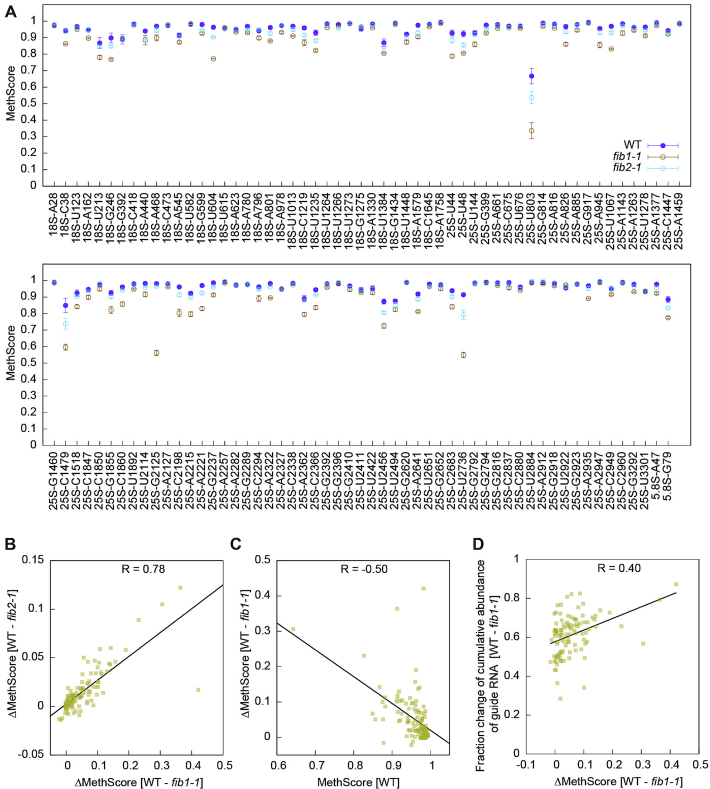
Methylation changes for individual sites in *fib* mutants. (**A**) MethScores are plotted as mean ± SD (*n* = 3 biological replicates) for all detected Nm in WT and two *fib* plants. (**B**) Correlation (Pearson's R) between MethScore changes in *fib1-1* and *fib2-1*. (**C**) Correlation between MethScore changes in *fib1-1* and MethScores in WT. (**D**) Correlation between MethScore changes and fraction changes of cumulated abundance of guide RNAs in *fib1-1*. Normalized TPM of all assigned guide RNAs for a methylation site were combined to calculate fraction of change between WT and *fib1-1*. The values in B–D are all based on means of *n* = 3 biological replicates.

Fibrillarin is encoded by the FIB1 and FIB2 genes in Arabidopsis (69). To characterize their role in RNA 2′-*O*-methylation, we profiled Nm in a T-DNA insertion mutant of FIB1 and FIB2, called *fib1-1* and *fib2-1*, respectively. The methylation of cytoplasmic rRNAs was significantly reduced in both *fib1-1* and *fib2-1* (Figures 6B, 7A). *fib1-1* caused a more pronounced decrease of methylation than *fib2-2* (mean change of MethScore = 0.054 versus 0.016), suggesting that FIB1 plays a bigger role in 2′-*O*-methylation.

The levels of C/D snoRNAs were globally reduced by 54% and 26% in *fib1-1* and *fib2-1*, respectively (Figure 6C–E). As a comparison, snRNAs accumulated normally (Figure 6D and E). The more pronounced reduction of C/D snoRNAs in *fib1-1* correlates with its greater decrease of rRNA methylation. As majority of snoRNA molecules are present in snoRNPs in cells, the level of snoRNAs should reflect the level of corresponding snoRNPs. Thus, deletion of one FIB gene globally inhibited the production of C/D snoRNPs, which in turn suppressed the 2′-*O*-methylation of rRNAs. The expression levels of H/ACA snoRNAs were also reduced by 26% and 22% in *fib1-1* and *fib2-1*, respectively, suggesting that the two types of snoRNAs were coordinately expressed.

An intron in the FIB1 and FIB2 genes harbors U60.1F and U60.2F snoRNAs, respectively (69). U60.1F was not expressed in *fib1-1*, whereas U60.2F was still expressed in *fib2-1* that contained a T-DNA insertion downstream of U60.2F (51) (Figure 6A). Consequently, methylation of 25S_G2125, the target of U60, was decreased in *fib1-1*, but unaltered in *fib2-1* (Figure 7A).

The degree of methylation reduction was variable for individual sites and correlated in two *fib* mutants (Figure 7B). The change of MethScore in *fib1-1* was moderately inversely proportional to MethScore (Pearson's *R* = –0.5) (Figure 7C). As an illustrative example, 25S_U803 with the lowest MethScore was one of sites showing the largest decrease of methylation in the *fib* mutants (Figure 7A). Similar correlation was observed previously in human cells upon knockdown of fibrillarin (23). Partial methylation sites are probably synthesized more slowly and hence more sensitive to reduced levels of enzymes in the *fib* mutants. Consistently, the change of MethScore for individual methylation site can be partially accounted for by the fraction of reduction in the cumulative abundance of guide RNAs (Figure 7D).

## DISCUSSION

We have experimentally determined the comprehensive and quantitative maps for Nm in Arabidopsis cytoplasmic, chloroplast and mitochondrial rRNAs and snRNAs. These maps of Nm would provide basic information for investigation of the function and biogenesis of Nm in these rRNAs. We expect that the identified Nm contain few false positive since the high and low score sites are well divided for all these RNAs. Nevertheless, some low degree methylation sites may be missed due to the technical limitation of RiboMeth-seq. Only one such site was identified for cytoplasmic rRNAs on the basis of the presence of guide RNA and the large decrease of methylation in the *fib* mutants. Mass spectrometry would be better suited for detecting low degree methylation sites (20,25). As a comparison, a RiboMeth-seq analysis of human rRNAs identified 106 of 112 sites, 14 sites of which have a MethScore below 0.75 (24,25). The six missed sites were mostly modified at low levels as showed by mass spectrometry.

We have compiled and cleaned the snoRNA list by collecting snoRNAs from various sources, standardization of snoRNA names, annotation of unexpressed snoRNAs, and addition of five novel snoRNAs. The high quality lists of Nm and C/D snoRNAs facilitated to build the snoRNA-target interaction network with improved completeness and accuracy and to identify outliers of Nm and snoRNAs. Guides have been assigned for 104/111 Nm in cytoplasmic rRNAs and 15/19 Nm in snRNAs according to the classic D+5 targeting rule. Nearly half of Nm were found to form extra pairs with snoRNAs, indicating that the extra pairing interaction between snoRNAs and target sites is prevalent besides yeast (18). The 11 unassigned sites could be guided by snoRNAs with multiple specificities or weak interaction, guided by unidentified snoRNAs, synthesized by stand-alone protein enzymes or merely experimental artifacts. Among the expressed C/D snoRNAs, SnoR4a/b, SnoR6.1/.2, most variants of SnoR43, SnoR105, SnoR106a/b, SnoR108, SnoR114, SnoR122 and SnoR165 appear to be orphan snoRNAs with no target on rRNAs and snRNAs (Supplementary Table S8).

Multiple specificities of the same guide sequence have been so far described only for yeast C/D snoRNAs (13,22,61). We have experimentally validated that Arabidopsis U24, SnoR58Y, SnoR29 and SnoR10 snoRNAs each target tandem sites at position 5 and 6 upstream of their box D'. The conservation of methylation sites and targeting pattern also suggest that Arabidopsis SnoR1 is the orthologue of yeast snR45 and both select two sites separated by one nucleotide. From the current limited examples, only the guide upstream of box D' is capable of targeting multiple substrates. The mechanism underlying multiple specificities of C/D snoRNA is unknown. It was suggested that the degenerated D' and C' motifs may form alternative structures, shifting the position of the substrate-guide duplex relative to the enzyme and the specificity of modification (61). Our findings demonstrate that C/D snoRNAs with multiple specificities are not limited to yeast and would aid study of the underlying mechanism.

Mitochondria and chloroplasts are organelles with their own translation machineries and believed to originate from bacteria by endosymbiosis. We have mapped five Nm in both mitochondrial and chloroplast rRNAs. Interestingly, four Nm occur at the identical positions in the two rRNAs and the fifth is located at adjacent sites in the A-loop. The correspondence between the Nm of mitochondrial and chloroplast rRNAs suggests that the equivalent methylation site is modified by the same or homologous enzyme that is likely encoded in the nuclear genome. The three sites located at the decoding center and the PTC are highly conserved, whereas the other two sites located at the subunit interface appear to be specific to Arabidopsis organelle rRNAs.

Arabidopsis has two FIB genes that are functionally redundant and expressed in all cells. *fib1-1* and *fib2-1* are null mutants according to the mRNA expression data, but they showed no obvious phenotype. The functional redundancy of FIB1 and FIB2 explains that their single mutants had more limited effect on the methylation of rRNA as compared to the *fib* mutants analyzed in human cells (23,27,30).

Finally, we discuss a few technical issues related to analysis of RiboMeth-seq data. (i) We found that a histogram diagram of MethScores for all analyzed sites is useful for assessing the specificity of measurement and determination of a threshold. MethScores were commonly reported only for identified methylation sites. (ii) Negative MethScores were set to zero in previous analysis of RiboMeth-seq data. We suggest that negative scores should be kept since they indicate that the queried site has a higher intrinsic hydrolysis rate compared to its neighbors. Moreover, the uncorrected negative score is required for correct calculation of MethScore change between samples and the level of methylation, which amounts to (MethScore of modified site - MethScore of unmodified site)/(1- MethScore of unmodified site). (iii) We have estimated that the average end count should be at least 15 for a reliable determination of MethScore. This value can guide the choice of sequencing depth in RiboMeth-seq experiments, in particular when less abundant RNAs are to be analyzed. By pooling multiple datasets, we have demonstrated that Nm can be identified for RNAs that are 100-fold less abundant than cytoplasmic RNAs.

## DATA AVAILABILITY

The RiboMeth-seq raw data have been deposited into the National Genomics Data Center (bigd.big.ac.cn) under GSA accession code CRA003263.

## Supplementary Material

gkab196_Supplemental_Files
