# Supplementary material for: Profiling of RNA ribose methylation in Arabidopsis thaliana
Source: Nucleic Acids Res. 2021 Mar 30;49(7):4104–19. doi: 10.1093/nar/gkab196 (PMC8053127; doi:10.1093/nar/gkab196)
Supplement: gkab196_Supplemental_Files [file gkab196_supplemental_files.zip › manu_AtNm_v8_supp.pdf]

Profiling of RNA ribose methylation in Arabidopsis thaliana

Songlin Wu, Yuqiu Wang, Jiayin Wang, Xilong Li, Jiayang Li and Keqiong Ye

Supplementary Materials

Figure S1-S4

Table S1-S2

Table S3-S13 (Sheets 1-11 in one Excel file).

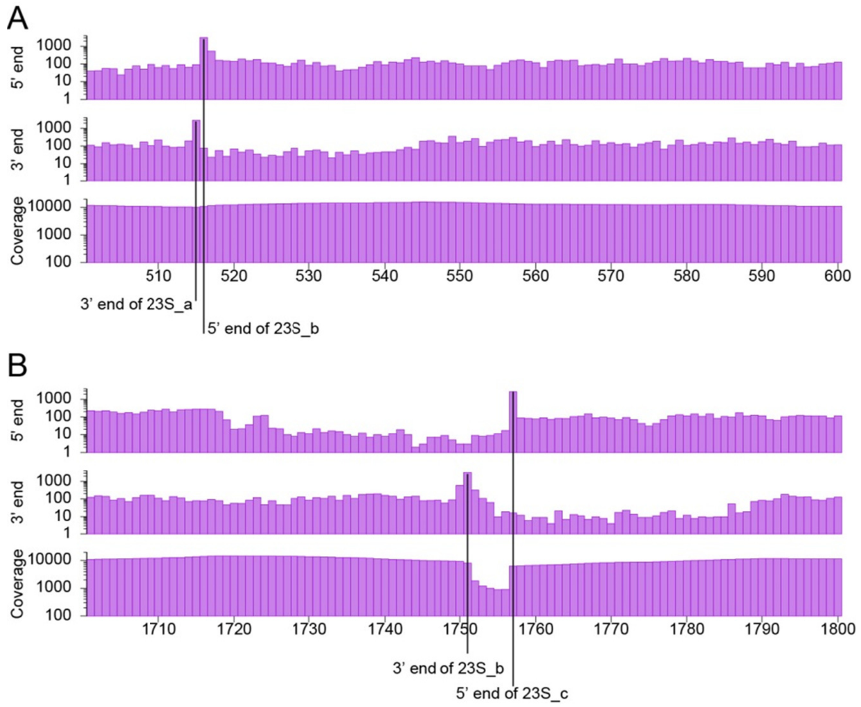

Figure S1. Chloroplast 23S rRNA is processed into three fragments.

(A-B) 5' and 3' end coverage and read coverage around the junctions between the 23S_a and 23S_b fragments (A) and between the 23S_b and 23S_c fragments (B).

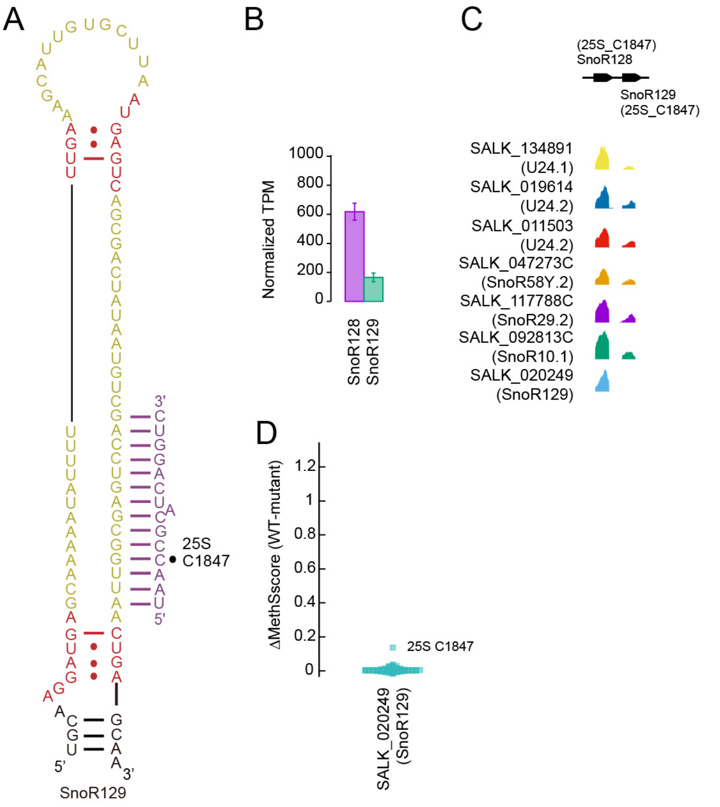

Figure S2. 25S C1847 is targeted by SnoR129.

(A) Secondary structure of SnoR129 and its interaction with its predicted target site of 25S rRNA. (B) Expression levels of the SnoR128 and SnoR129 variants in the WT plant. (C) Read coverage of snoRNAs in the analyzed T-DNA mutants normalized against 18S rRNA. The gene organization of snoRNA clusters and the predicted targets of C/D snoRNAs are displayed. (D) Beeswarm plot showing MethScore changes between WT and mutant plants for all identified Nm in cytoplasmic rRNAs. The sites with prominent reduction of methylation are labeled. MethScores were calculated as means of n=3 independent samples for wild-type and n=1 for the SnoR129 mutant.

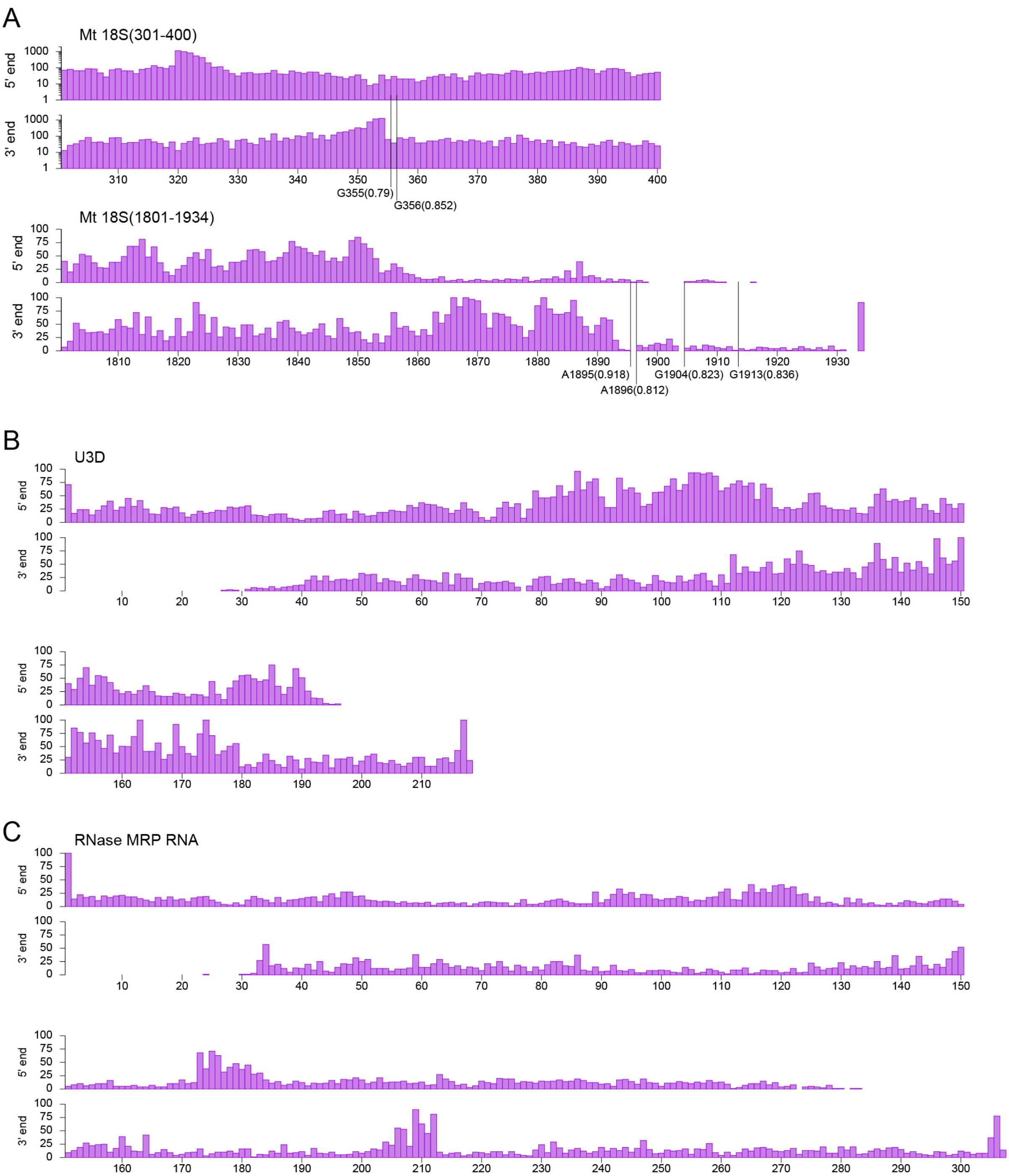

Figure S3. Tracks of 5' and 3' end counts in RiboMeth-seq analysis of other abundant RNAs.

(A) Several artificial high score sites in mitochondrial 18S rRNAs due to uneven or low end coverages. The 5' and 3' end counts are plotted in two panels for nt 301-400 and nt 1801-1934. Note that the y axis is shown in the log scale for nt 301-400. (B-C) No methylation site was found in U3 snoRNA (B) and RNase MRP RNA (C).

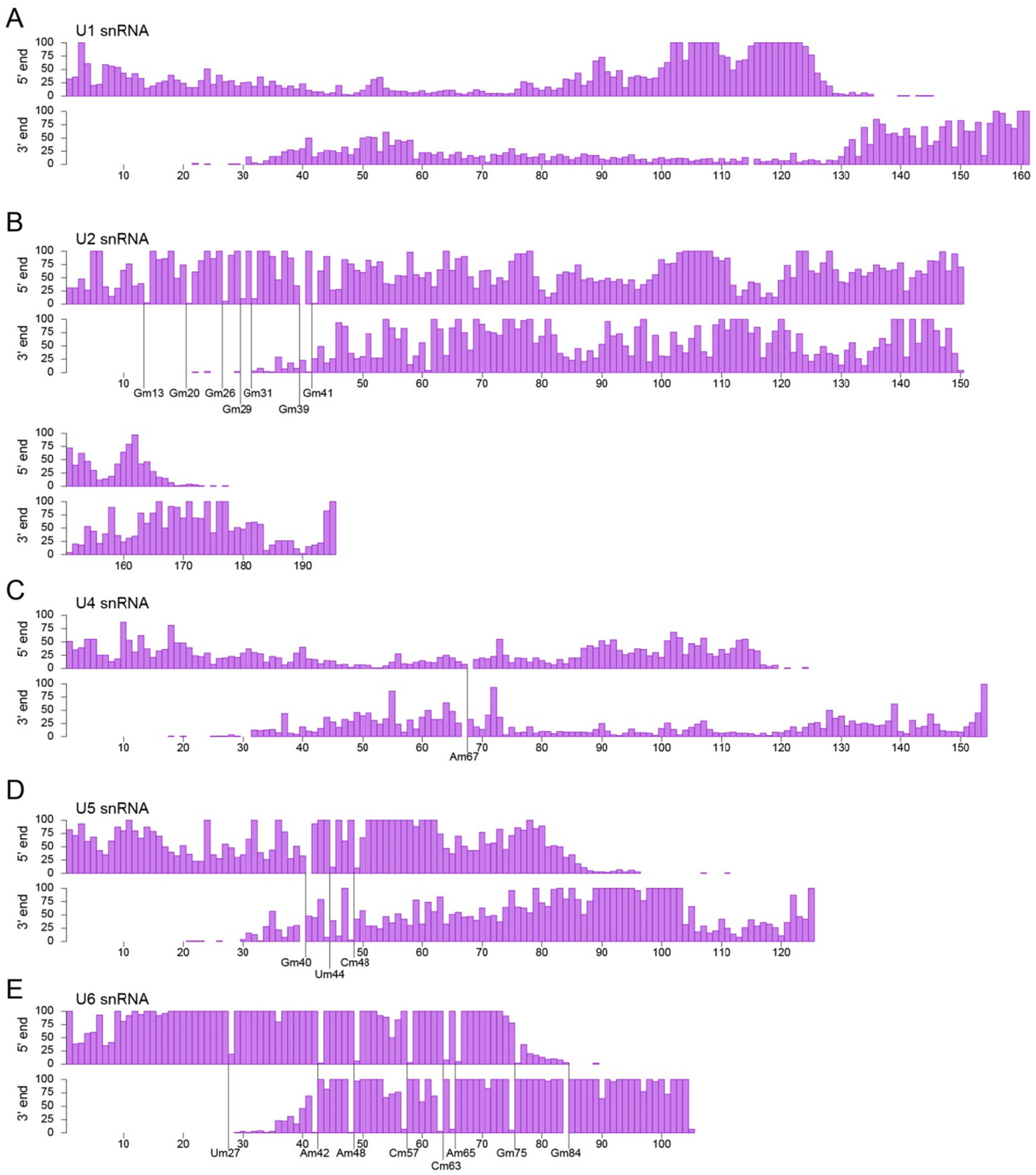

Figure S4. Tracks of 5' and 3' end coverage in RiboMeth-seq analysis of snRNAs.

(A) U1 snRNA. (B) U2 snRNA. (C) U4 snRNA. (D) U5 snRNA. (E) U6 snRNA. The data with end count > 100 are out of scale. A methylation site at position i is marked by a line between positions i and i+1and would show a lack of 3' end count at position i and a lack of 5' end count at position i+1. The 5' end count is shifted upstream by 1 position and combined with the 3' end count for calculation of MethScore.

Table S1. Genotyping primers

Strain	Name	Sequence (5'-3')
CS858544	*fib1-1*-LP	CACCGCACATCTCTCTCCTTTG
	fib1-1-LP	GACTTACAGGGCCAACGAGGTC
	LBb1	GCGTGGACCGCTTGCTGCAACT
SALK_093373C	*fib2-1*-LP	AGTTTCTGCGTGAGCTTTTTG
	fib2-1-RP	CTTCTCTGATGTTGCTCAGCC
	LBb1.3	ATTTTGCCGATTTCGGAAC
SALK_134891	SALK_134891-LP	AATCAACGGATGCCTAAGGAC
(U24.1)	SALK_134891-RP	GAATTCAAAGGGAATCCGAAC
SALK_011503	SALK_011503-LP	CGTCATTAAGCTGAGCTTTCG
(U24.2)	SALK_011503-RP	TAAGGGAATCATTGGGAATCC
SALK_019614	SALK_019614-LP	CGTCATTAAGCTGAGCTTTCG
(U24.2)	SALK_019614-RP	TAAGGGAATCATTGGGAATCC
SALK_047273C	SALK_047273-LP	GCTAGCATTTGAATGAGACGG
(SnoR58Y.2)	SALK_047273-RP	TCCCAGAGATATAACTCCGGG
SALK_117788C	SALK_117788-LP	TGTAACTTCACTTGGAGATGCAC
(SnoR29.2)	SALK_117788-RP	AGCGTAATTTCCATCGACATG
SALK_092813C	SALK_092813-LP	GAGTGGACCAGATGCTTGAAG
(SnoR10.1)	SALK_092813-RP	GGATAGAAACGAAGCCCAAAC
SALK_020249	SALK_020249-LP	GTAGCCCATTTTACGCCTACC
(SnoR129)	SALK_020249-RP	TCTTCGCTAGTGGACATGTCC

Table S2. Oligos used in RiboMeth-seq

Oligonucleotide	Sequence[a]
3' adaptor	5'-rAppTNNNTAGNNNTGGAATTCTCGGGTGCCAAGG-NH_2-3'
5' adaptor 1	5'-ACACGACGrCrUrCrUrUrCrCrGrArUrCrUrNrNrNrCrGrNrNrNrU-3'
5' adaptor 2	5'-ACACGACGrCrUrCrUrUrCrCrGrArUrCrUrNrNrNrArUrNrNrNrC-3'
Reverse transcription primer	5'-GCCTTGGCACCCGAGAATTCCA-3'
P7	5'-CAAGCAGAAGACGGCATACGAGAT[i7]GTGACTGGAGTTCCTTGGCACCCGAGAATTCCA-3'
P5	5'-AATGATACGGCGACCACCGAGATCTACAC[i5]ACACTCTTTCCCTACACGACGCTCTTCCGATCT-3'

[a]Deoxynucleotides are represented by single letter and ribonucleotides are marked with "r". "N" denotes random sequence. Barcodes are underlined. i7 and i5 refer to index sequences of 6 nt and 5 nt, respectively.

Table S3. Reference RNA sequences.
Column A-F: Location of reference genes in the BED format.

Table S4. MethScore of cytoplasmic rRNAs and previously predicted or verified sites.
Column I-J: Abbreviation of reference: (1) Barneche et al., 2001; (2) Brown et al., 2001; (3) Qu et al., 2001; (4) Marker et al., 2002; (5) Chen and Wu, 2009; (6) Kim et al., 2010; (7) Wang et al., 2014; (8)Qu et al., 2015; (9) Cheng et al., 2017; (10) Kruszka et al., 2003; (D1) Plant snoRNA database; (D2) SnoPY database.
Column K-U: MethScores for three repeats of WT, *fib1-1* and *fib2-1* and one measurement of *hid1* and *hid2*.

Table S5. MethScores of chloroplast rRNAs.
Column G-Q: MethScores for all samples.

Table S6. MethScores of mitochondrial rRNAs.
Column F: MethScores from the pooled datasets.

Table S7. MethScores of snRNAs.
Column E: Artificial high score sites are labeled as fake.
Column F: MethScore from the pooled datasets.

Table S8. SnoRNA genes and expression.
Column A: Index for sorting
Column B-G: Location of snoRNA genes in the BED format. The start coordinate is 0-based and the end coordinate is 1-based.
Column H-I: Predicted targets for the D' and D guide.
Column L: A snoRNA gene is considered not expressed if the average normalized TPM of three WT samples is less than 20.
Column O-Y: Normalized TPM for all samples.

Table S9. Novel snoRNAs identified in this study.
Column A-F: Location of snoRNA genes in the BED format.

Table S10. SnoRNA genes predicted by Qu et al 2015.

Table S11. Nm in cytoplasmic rRNAs and their predicted guides.
Column E: Average MethScore of three WT samples.
Column G: Equivalent modification site in yeast rRNAs
Column H: The responsible C/D guide snoRNA or protein enzyme in yeast.
Column L: Length of primary pairs was measured by excluding pairs that overlap with box D/D'.

Column M-R: Major and extra pairs are displayed for one of snoRNA variants. Ranges of residues involved in base pairing interaction are shown in brackets for rRNA and snoRNA. Number of base pairs is shown in parentheses.
Column S-AC: Cumulative normalized TPM of guide RNAs for all samples.

Table S12. Nm in snRNAs and their predicted guides.
Column K-U: Cumulative normalized TPM of guide RNAs for all samples.

Table S13. Correspondence of Nm in Arabidopsis, yeast and human cytoplasmic rRNAs.
Column I-L: MethScores of experimentally detected Nm. ND, Not determined.
Column M: Methylation levels of human Nm detected by mass spectrometry (Taoka et al 2018)
Column N: A=Arabidopsis, Y=Yeast, H=Human
